# Risk Factors for Motorcycle-related Severe Injuries in a Medium-sized City in China

**DOI:** 10.3934/publichealth.2016.4.907

**Published:** 2016-11-08

**Authors:** Lili Xiong, Yao Zhu, Liping Li

**Affiliations:** 1 Hunan Province Maternal and Children Health Care Hospital, 53 Xiang Chun Road, Changsha, Hunan Province, China 410000; 2 Injury Prevention Research Center, Shantou University Medical College, 22 Xin Ling Road Shantou, Guangdong Province, China 515041

**Keywords:** motorcycles, injuries, risk factors

## Abstract

**Background:**

Motorcycle vehicles are frequent in China, especially in the small and medium sized cities. Road traffic collisions involving motorcycles often result in severe injuries. We aimed to identify risk factors for severe injuries in inpatients sustaining motorcycle collisions.

**Methods:**

Patients with road traffic injuries involving motorcycles who presented to the neurosurgery and orthopedic departments of three major comprehensive hospitals in Shantou city were reviewed from October 2012 to June 2013. Data from 349 patients was investigated. Crash and injury characteristics were documented by interviewing patients, their family members, and their doctors. Binary logistic regression was used to determine risk factors for severe injuries.

**Results:**

There were 253 males (72.49%) and 96 females (27.51%), with a male to female ratio of 2.64:1. The mean age was 38.21±17.32 years. One-hundred and fifty patients were in the severe injury group with a mean injury severity score (ISS) of 15.34±9.13. The simple and multiple logistic model showed that males, lack of safeguards, morning and night hours, non-urban areas, collision of a motorcycle with a cycle, ambulance transportation to hospital, admission to a neurosurgery department, lack of traffic control, unobstructed traffic, and poor visibility were all the risk factors.

**Conclusions:**

This research highlights some problems: less helmet wearing in motorcyclists and cyclists, rural injuries being more serious than urban ones, and head injuries being the main diagnosis in severe injuries. The result of this research is predictable. If the safety equipment is required to be used, such as helmets, and the traffic environment is improved, such as traffic flow, medical resources to injuries and deaths is seasonable, then traffic safety will be improved and accidents will be reduced.

## 1. Introduction

Road traffic injuries are a serious public health problem. According to the World Health Organization report in July 2013, road traffic injuries have increased from 11^th^ in 2000 to 9^th^ in 2011 in cause of death, and will be 5^th^ by 2030 [1]. Although injuries due to road-traffic incidents impose a substantial burden globally, the burden is greatest in low-and middle-income countries [2], particularly in China [3],[4]. Motorcycle-related injuries account for the majority of injuries and deaths related to road traffic in China [5],[6]. Although motorcycles have been restricted from travelling in large cities such as Beijing, Shanghai, and Shenzhen due to their negative effects on society from pollution, traffic congestion and other reasons, motorcycles are used extensively in small and medium sized cities and rural areas, due to ease, convenience, and affordability. The total number of motorcycles owned in China increased from 2.5 million (23% of total motorized vehicles) in 1987 to 50 million (70%) in 2002, to 70 million (54%) in 2005, and to over 100 million in 2010 (46%) [7],[8]. Unfortunately, road traffic injuries involving motorcycles often result in severe injuries [9]–[12]. For example, motorcycle accidents resulted in 26,200 deaths and 157,500 injuries in 2005, and 11,070 deaths and 59,455 injuries in 2011 in China [6],[8]. The proportion of fatalities and injuries in motorcycle collisions is even higher in some medium-sized cities where motorcycle use is more common, for example over 50% of total traffic injuries in Shantou city [13].

Many risk factors are associated with the incidence of severe injuries in motorcycle crashes. These include demographic characteristics (age, gender, helmet wearing, behavior), environmental characteristics (traffic flow, traffic control, visibility condition, weather), and crash characteristics (time, rural or urban location, type of collision) [14]–[25]. Risk factors related to severe motorcycle injuries have been investigated worldwide and in larger cities; but the relevant research in medium cities in China has not been well studied. We aimed to identify contributing factors related to severe injuries for inpatients involving motorcycle-related injuries, and to provide references to prioritize programs that would prevent and reduce such injuries.

## 2. Methods

Shantou is a medium-sized city in China, with a population of approximately 5 million habitants at the time of the study, and is one of the five special economic zones and an important port city in the southern region. There are three first-class public comprehensive hospitals, which have a total of 5,600 beds and treat more than 95% of severe trauma patients in the region.

Data were collected prospectively over ten months from October 2012 to July 2013. Patients with motorcycle traffic injuries who were admitted to the neurosurgery and orthopedic departments of the three hospitals were included if their hospital stay was greater than 24 hours and they survived, regardless of injury severity. We have established cooperative relations with the six departments. When patients meet the requirements, the head nurse will inform us two days after the patient is in the hospital. In this study, a motorcycle was defined as a two-wheeled motor vehicle; moped injuries were not addressed separately from motorcycle injuries. Motorcycle-related injuries were defined as traffic accidents involving a motorcycle or two. This study was approved by the ethics committee of the medical college, Shantou University, the first and second affiliated hospital of Shantou University, and Shantou center hospital. Informed written consent was obtained from the patients, their family, or caregivers prior to study enrollment.

Demographic information, environmental, and crash characteristics were acquired from patients, families, or their caregivers, while injury severity scores were obtained from their doctors. Demographic information included age, gender, safeguards, and road users. Environmental factors included weather, visibility conditions, traffic flow conditions, and manner of traffic control in the area of the crash site. Crash characteristics included the time and location of the crash, type and direction of the collision, unsafe behavior, methods of transport to hospital after the crash, and the admitting department. The dependent variable of injury severity score (ISS) was dichotomous to severe injuries with an ISS of ≥16 and non-severe injury with an ISS of <16.

### Statistical Analysis

Data were analyzed using STATA software, version 11.2. A descriptive analysis was conducted for each variable. The frequency of distribution was created for patients, counted by the road users among age groups, and patients counted by the crash types among road users in the severe and non-severe injury groups. The chi-squared test was used to define factors affecting injury severity. Variables with a *p*-value less than 0.05 were entered into the statistical model. Correlation analyses between the affecting variables were performed. In the case when one variable correlated with another with a correlation value of 0.5 or above, only one variable remained in the model based on the best model choice. Then, simple logistic regression was used to get crude odds ratios (OR) and 95% confidence intervals (95%CI). Finally, multiple logistic regression was conducted to estimate the OR of severe injury for the selected variables, adjusting for all other factors in the model.

## 3. Results

### 3.1. Demographic Characteristics

**Figure 1.**
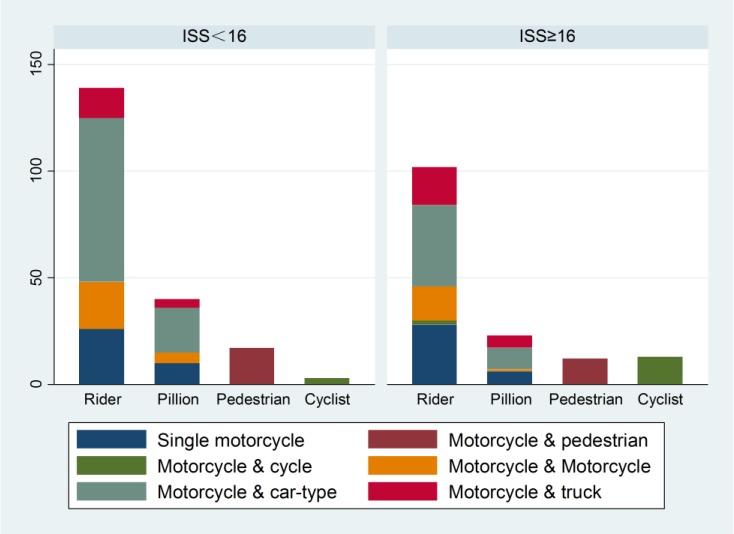
Frequency of patients counted by road users among age groups. The majority of patients were distributed in the 15–29 years (37.54%), 45–59 years (25.21%) and 30–44 years (21.20%) age groups. The highest frequencies of riders and pillions were distributed in the 15–29 years (39.83% and 49.21%) age group. The highest frequency of pedestrians and cyclists was distributed in above 60 years (41.38% and 31.25%) age group.

There were 401 eligible patients over a ten month period. We analyzed a total of 349 patients who took part in the survey eventually. The mean injury severity score (ISS) was 15.34 with a standard deviation of 9.13. One hundred and fifty patients were classified in the severe injury group and 199 patients were in the non-severe injury group. The patients' age ranged from 3 to 83 years with a mean and standard deviation of 38.21±17.32 years. There were 253 males (72.49%) and 96 females (27.51%), with a male to female ratio of 2.64:1 (Table 1). Motorcyclists are often injured in crashes involving pedestrians or cyclists. The most frequent participant role in road traffic of our cohort was the motorcycle rider (n = 241, 69.05%), pillion passenger (n = 63, 18.05%), pedestrian (n = 29, 8.31%), and cyclist (n = 16, 4.58%). There was a statistically significant correlation between participant role and age group (χ^2^ (12) = 68.57, *p* = 0.00). Almost all motorcycle riders and passengers were distributed in 15–29 years (39.83% and 49.21%). Pedestrians and cyclists were distributed in the above 60 year age group (41.38% and 31.25%, Figure 1).

**Table1.** Demographic characteristics between severe and non-severe injury groups.

Variables	ISS ≥ 16	ISS < 16	χ^2^	*p*-value
Gender			6.17	0.01
Female	31 (20.67)	65 (32.65)		
Male	119 (79.33)	134 (67.34)		
Age groups			1.93	0.75
≤ 14	4 (2.67)	6 (3.02)		
15–29	61 (40.67)	70 (35.18)		
30–44	33 (22.00)	41 (20.60)		
45–59	33 (22.00)	55 (27.64)		
≥ 60	19 (12.67)	27 (13.57)		
Safeguard *			25.68	0.00
Yes	14 (9.33)	64 (32.16)		
No	136 (90.67)	135 (67.84)		
Road users			10.71	0.01
Motorcycle riders	102 (68.00)	139 (69.85)		
Passengers	23 (15.33)	40 (20.10)		
Pedestrians	12 (8.00)	17 (8.54)		
Cyclists	13 (8.67)	3 (1.51)		

* Safeguards include mainly helmets for motorcycle riders, passengers, and cyclists.

Gender, safeguard practices, and road users were associated with the dependent variable (Table 2). Males were associated with severe injury (OR = 1.86, 95% CI 1.14–3.05) in the simple logistic model, though there was no statistical significance found in the adjusted model (Table 3). Lack of safeguard precautions was linked to severe injury in the simple logistic model (OR = 4.61, 95% CI 2.46–8.61) and adjusted logistic model (OR_j_ = 3.32, 95% CI 1.57–7.04).

### 3.2. Environmental Characteristics

Light conditions, traffic flow, and traffic control conditions were the three environmental variables related to severe injury outcome, whereas other road environmental factors such as weather conditions showed no statistical significance (Table 2). In the adjusted logistic model, three environmental factors did not contribute to severe injury.

### 3.3. Crash Characteristics

**Figure 2.**
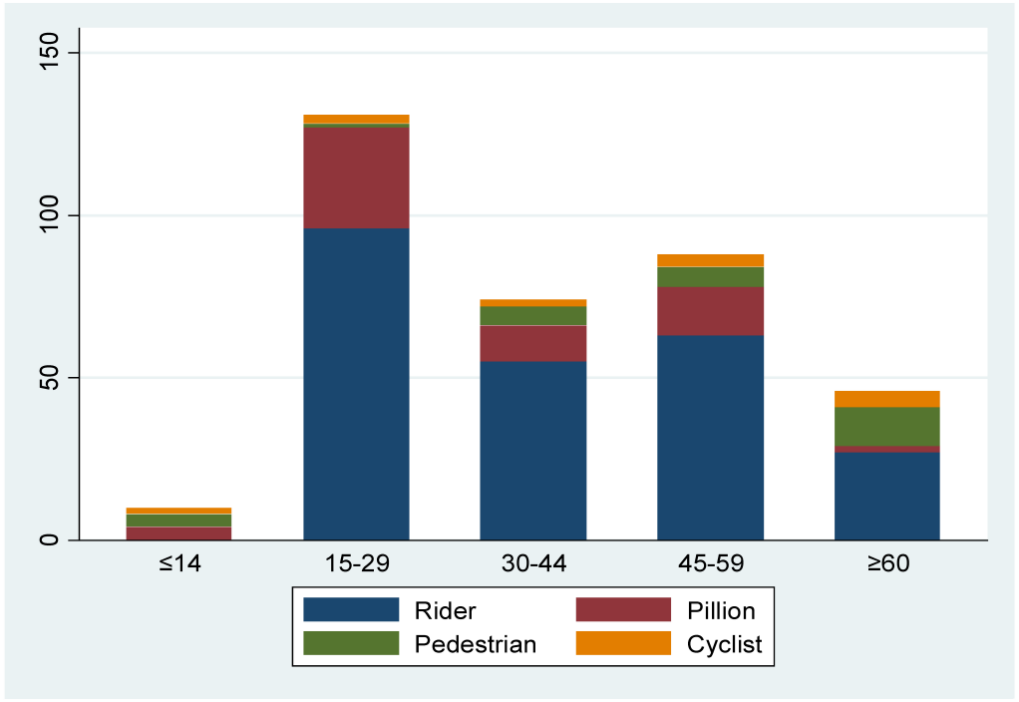
Frequency of crash types by road users and injury severity. The majority of patients in non-severe and severe injury groups were distributed as riders (69.85% and 68%); the main crash type in the non-severe and severe injury groups were both a collision of a motorcycle with a car-type (49.25% and 32.00%) and a single motorcycle crash (18.09% and 22.67%). The majority of crash types of a motorcycle with a cycle were distributed in the severe injury (83.33%) group, while collisions of a motorcycle with a car were in the non-severe injury group (67.12%).

The most frequent crash types among patients were collisions of a motorcycle with a lightweight vehicle (n = 146, 41.83%) and single motorcycle crashes (n = 70, 20.06%). There was a statistical significant difference between crash types and injury severity (χ^2^ (5) = 22.74, *p* < 0.001) and the same association was found between road users and injury severity (χ^2^ (3) = 10.71, *p* = 0.01). The distribution of patients counted by crash types among road users in the non-severe and severe injury groups is shown in Figure 2. The majority of crash types in the non-severe and severe injury groups were both a collision of a motorcycle with a light vehicle (49.25% and 32.00%) and a single motorcycle crash (18.09% and 22.67%). The majority of collisions of a motorcycle with a cycle were distributed in the severe injury group (83.33%), while collisions of a motorcycle with a light vehicle were distributed in the non-severe injury group (67.12%). Crashes of a motorcycle with a cycle occurred for victims of cyclists (n = 13, 81.25%) and resulted in severe injury in the 60 years age group.

Among the five levels of crash time in a 24-hour period, the times between 20:00 and 24:00 had the highest percentage (n = 40, 26.67%) of severe injury, while the period between 10:00 and 16:00 had the highest percentage of non-severe injury (n = 66, 33.17%). More patients involved in severe injury were in a rural location (n = 57, 38%) compared with those involved in non-severe injury (n = 70, 35.18%). The proportion of victims using the ambulance on the way to the hospital (n = 121, 80.67%) in the severe injury group was higher than those in the non-severe injury group (n = 135, 67.84%) (χ^2^ (1) = 7.20, *p* = 0.01). Two hundred and eighteen (62.46%) patients were admitted to the neurosurgery department, while the remaining 131 (37.54%) patients were admitted to the orthopedics department.

Collisions of a motorcycle with a cycle were associated with severe injury in the simple logistic model (OR = 7.26, 95% CI 2.06–25.61) and adjusted logistic model (OR_j_ = 94.75, 95% CI 15.70–57.19). The times of 0:00-6:00 (OR = 2.45, 95% CI 1.40–4.27) and 20:00–24:00 (OR = 2.48, 95% CI 1.47–4.47) were associated with severe injury. Location was not related to severe injury (OR = 0.48, 95% CI 0.31–0.74). Ambulance transportation to the hospital was linked to severe injury (OR_j_ = 1.99, 95% CI 1.02–3.85). Compared with the orthopedics department, patients in the neurosurgery department had an increased chance of severe injury of about nine times (OR_j_ = 9.28, 95% CI 4.60–18.74).

**Table 2.** Crash and environmental characteristics between severe and non-severe patients.

Variables	ISS ≥ 16	ISS < 16	χ^2^	*p*-value
Time of crash			34.26	0.00
0:00–6:00	39 (26.00)	25 (12.56)		
6:00–10:00 & 16:00–18:00	29 (19.33)	63 (31.66)		
10:00–16:00	24 (16.00)	66 (33.17)		
18:00–20:00	12 (8.00)	8 (4.02)		
20:00–24:00	40 (26.67)	26 (13.07)		
unknown	6 (4.00)	11 (5.53)		
Location of crash			18.16	0.00
Urban	56 (37.33)	110 (55.28)		
Rural	57 (38.00)	70 (35.18)		
Unknown	37 (24.67)	19 (9.55)		
Behavior			1.79	0.18
Unsafe *	5 (3.33)	13 (6.53)		
Safe	145 (96.67)	186 (93.47)		
Type of collision			22.74	0.00
Single motorcycle	34 (22.67)	36 (18.09)		
Motorcycle-pedestrian	12 (8.00)	17 (8.54)		
Motorcycle-bicycle	15 (10.00)	3 (1.51)		
Motorcycle-motorcycle	17 (11.33)	27 (13.57)		
Motorcycle-light vehicle	48 (32.00)	98 (49.57)		
Motorcycle-heavy vehicle	24 (16.00)	18 (9.05)		
Transport to hospital			7.20	0.01
Ambulance	121 (80.67)	135 (67.84)		
Other means	29 (19.33)	64 (32.16)		
Department admitted			73.16	0.00
Neurosurgery	132 (88.00)	86 (43.22)		
Orthopedics	18 (12.00)	113 (56.78)		
Visibility			4.32	0.03
Day/street lamps	116 (77.33)	171 (85.93)		
Without street lamps	34 (22.67)	28 (14.07)		
Traffic condition			5.49	0.02
Unobstructed	139 (92.67)	168 (84.42)		
Obstructed	11 (7.33)	31 (15.58)		
Traffic control*			4.19	0.04
Yes	30 (20.00)	59 (29.65)		
No	120 (80.00)	140 (70.35)		
Weather condition			0.01	0.91
Sunny	131 (87.33)	173 (86.93)		
Rainy	19 (12.66)	26 (13.07)		

* Unsafe behavior includes alcohol consumption, speeding, running a red light, using a mobilephone, violating traffic, etc. Traffic control refers to traffic light, traffic marking line, policeman direction, etc.

**Table 3.** Odds Ratio by risk factors in simple and multiple logistic models.

	Risk factors	OR (95% CI)	OR_j_ (95% CI)
Demographic	Age	(RE: ≥ 60years)	
	≤ 14years	0.88 (0.24–3.19	0.82 (0.04–16.53)
	15–29years	1.26 (0.82–1.96)	0.50 (0.21–1.22)
	30–44years	1.09 (0.65–1.82)	0.39 (0.15–1.01)
	45–59years	0.74 (0.45–3.05)	0.73 (0.29–1.88)
	Gender	(RE: Female)	
	Male	1.86 (1.14–3.05)	1.50 (0.74–3.05)
	Road users	(RE: Cyclists)	
	motorcycle riders	0.92 (0.58–1.45)	5.10 (3.42–7.58)
	Passengers	0.72 (0.41–1.27)	4.75 (2.70–8.35)
	Pedestrians	0.93 (0.43–2.02)	9.95 (3.99–24.84)
	Safeguard	(RE: Yes)	
	No	4.61 (2.46–8.61)	3.32 (1.57–7.04)
Crash	Time of the crash	(RE: Unknown)	
	0:00–6:00	2.45 (1.40–4.27)	4.01 (0.53–30.58)
	6:00–10:00 & 16:00–18:00	0.72 (0.56–0.93)	1.01 (0.36–2.81)
	10:00–16:00	0.73 (0.61–0.87)	0.91 (0.47–1.77)
	18:00–20:00	0.37(0.31–0.44)	0.45 (0.23–2.07)
	20:00–24:00	2.48 (1.47–4.47)	4.34 (0.78–28.78)
	Location of the crash	(RE: Others/unknown)	
	Urban	0.48 (0.31–0.74)	0.52 (0.24–1.14)
	Rural	1.06 (0.85–1.32)	0.94 (0.63–1.40)
	Type of collision	(RE: Motorcycle * pedestrian/Motorcycle * heavy vehicle)	
	Single motorcycle	1.33 (0.78–2.25)	0.30 (0.10–0.89)
	Motorcycle-bicycle	7.26 (2.06–25.61)	94.75 (15.70–57.19)
	Motorcycle-motorcycle	0.81 (0.43–1.56)	0.17 (0.05–0.53)
	Motorcycle-light vehicle	0.48 (0.31–0.75)	0.41 (0.14-1.19)
	Transport to hospital	(RE: Other transportation)	
	Ambulance	1.98 (1.20–3.27)	1.99 (1.02–3.85)
	Department admitted	(RE: Orthopedics)	
	Neurosurgery	9.64 (5.46–17.00)	9.28 (4.60–18.74)
Environmental	Visibility	(RE: Day/with street lamps)	
	Without street lamps	1.79 (1.03–3.11)	1.54 (0.73–3.25)
	Traffic condition	(RE: Obstructed)	
	Unobstructed	2.33 (1.13–4.81)	1.98 (0.67–5.92)
	Traffic control	(RE: Yes)	
	No	1.69 (1.02–2.79)	0.89 (0.44–1.78)

* Goodness-of-fit of multiple logistic model: χ^2^ (323) = 407.15, *p* = 0.001; Number of observations = 349; Log pseudo likelihood = −161.67; Wald chi2 (22) = 328.31, prob = 0.00; Pseudo R^2^ = 0.32. OR_j_ (adjust odds ratio): adjusted for all other risk factors in the binary logistic model; RE: reference group.

## 4. Discussion

We found that risk factors involved in severe motorcycle injuries included male gender, lack of a safeguard, the times of 0:00–6:00 and 18:00–24:00, non-urban locations, collision of a motorcycle with a cycle, use of an ambulance for transport to the hospital, admission to the neurosurgery department, lack of traffic control, unobstructed traffic conditions, and poor visibility.

Most of riders were distributed in the young age group. Young riders are associated with risk taking behaviors and inexperience, which increase their risk of being involved in motorcycle injuries [25],[26]. Age was not associated with injury severity in this study. However, as older people tend to have reduced perceptual ability and sensation, their hospital course is more likely to be challenged by complications contributing to their poorer outcomes [27]. Additionally, the relationship between age and severity of motorcycle crashes is controversial. Some studies suggest that older motorcyclists were more likely to sustain severe injuries [28],[29], while other reports showed a strong and consistent relationship between increasing age and decreasing injury risk [30]. We found that male gender was associated with severe injury. Most injured persons in motorcycle crashes were typically of the lowest legal age groups, male riders, and demonstrating specific patterns of youth behaviors, such as aggression, negligence of traffic regulations, and lack of helmet use [16].

The odds of being in a severe injury during the times of 0:00–6:00 and 20:00–24:00 were 2.45 and 2.48, respectively. In general, injuries after midnight have been found to be the most severe because of increased speeds and higher impacts [18],[20]. Nighttime crashes may be related to visibility, such as riding without streetlamps, which has been related to severe injury [19],[20],[24]. In the conditions of no light, the most frequent crash type was a collision of a motorcycle with a car (35.48%). Pai et al. found that the right-of-way violation of motorists in a collision of a motorcycle with an automobile was more likely to occur under diminished light conditions [19]. Haque et al. found that reduced conspicuity of motorcycles at night was particularly hazardous for several situations, such as merging and diverging on expressways, and turning maneuvers at intersections [23]. Thus, the effect of visibility to severe injury may be offset by crash time or location, resulting in statistical significance in the crude analysis but vice versa in the adjusted model.

Traffic control, not only referring to signalization, may play an important role in road traffic injury. Under un-signalized conditions, motorcycle crashes were about 11.9, 37.5, 2.6 and 2.3 times higher than signalized conditions at three-legged intersections, on single lane roads, and on curb and median lanes, respectively [31]. We had not explored the risk of severe injury under the situation of signalization in different road types; our study showed that without traffic control (such as traffic signals), the direction of traffic police increased severe injuries by 1.7 times. However, this variable did not reveal its association in the adjusted model. The same happens to the variable of traffic flow. Traffic flow was associated with severe injury in the simple logistic model, but its effect had no significance in the adjusted model. In the London charging zone, the congestion charge reduces the total number of car accidents, but leads to an increase in two-wheeled vehicle accidents [32]. We found a negative relationship between traffic flow (congestion) and injury severity of motorcycle casualties [33]. The flow of traffic may be influenced by crash time or location.

Crash location was one of the major influential factors affecting the probability of severe injury. The prevalence of severe or fatal injuries in rural areas was relatively higher than in urban locations in different traffic environments; rural roads were associated with lack of helmet use, drunk driving, and speeding [15],[17],[20],[21],[34]. Regarding safeguard practices, especially the use of helmets, we found that lack of a safeguard resulted in a higher risk of severe injury. Only 29.46% and 11.11% of motorcycle riders and passengers used a helmet. It has been widely established that helmets can reduce the risk of fatal injuries by 42% and the risk of head injury by 69% for motorcyclists [35]. In March 1988, the helmet law was made mandatory for all motorcycle riders in China, and it was enacted in May 2004. However, the proportion of helmet use in motorcycle riders and passengers was 66% and 29% in a roadside observation in 2006 in Shantou city [36]. Enforcement and education prevention need to be strengthened with respect to helmet use, especially in rural areas [37]. We did not observe helmet use in cyclists. A systematic review showed that helmet use results in a 63–88% reduction in the risk of head injuries for all ages of bicyclists [38]. However, there is a debate over bicycle helmet use in China, because this requirement could have a negative effect, such as unsafe behavior [39]. There is no national cyclist helmet law in China.

Unsafe behavior is not independently associated with a risk of severe injury and only 4.3% of the cohort involved alcohol consumption. Differently, kung fu tea is very popular and has become a local custom instead of drinking alcohol in other places. However, it is often reported that road traffic victims in the hospital had consumed alcohol prior to their accidents [15],[40]. Motorcycle riders are more vulnerable than other motor vehicle drivers because of the effect of alcohol on balance, motor coordination, and judgment, and because more basic skills are required to operate an unstable vehicle [41],[42]. Alcohol use is a significant risk factor for pedestrians to cross the street unsafely, and to be struck by motor vehicles [43]. Risky behavior has been consistently recognized as a key contributor to road crashes and many studies have observed its association with severe injury, such as speeding motorcycle riders, mobile phone use by pedestrians, and red light infringement by cyclists [25],[44]–[46]. In our study, weather conditions were not related to injury severity although motorcycle riding was heavily influenced by weather. 87.11% of all crashes occurred in sunny weather, which is common in the city. However, weather was not found to contribute to accidents and has been reported to be less influential compared to other factors such as safeguard use and types of collision [22],[31],[47].

Regarding types of crashes, motorcycle crashes involving cycles contributed to injury severity. The explanation may be that all victims in collisions of a motorcycle with a cycle were cyclists distributed in the older age group. Second, most of patients in collisions of a motorcycle with a car were distributed in the non-severe injury group. Third, the highest frequency of crash types were collisions of a motorcycle with a car and single motorcycle crashes. So far, no consistent conclusion has been made about the severity of single motorcycle crashes and multi-vehicle crashes involving a motorcycle [48]–[51]. We did not distinguish the specific crash model from single motorcycle injuries, which included barrier collisions, fixed object collisions, overturn collisions, and rollovers. Different crash models had different traits in single motorcycle crashes [48],[49].

In the three hospitals, most victims injured in road traffic were sent to the neurosurgery and orthopedics departments; each hospital has five to eight subordinate departments. Additionally, head injuries were the most frequent in fatal motorcycle crashes and extremity injury was the most common injury in all motorcycle crashes [35],[42],[52],[53]. In our study, 66.76% of patients and 95% of severe trauma patients suffered head injuries. Head injury was the leading cause of death in motorcycle crashes; so patients admitted to the neurosurgery department had a higher injury severity than patients in the orthopedics department. When faced with serious traffic accidents, people often call the emergency telephone number (120) and an ambulance. Thus, ambulance transportation was linked with severe injury.

Previous studies similar to this topic adopted police report data, hospital data, or both. Police and hospital records used in motorcycle injury studies were often incomplete, in that they usually over-represent severely injured subjects [54]–[58]. Considering that police report data does not supply information such as alcohol consumption and risky behavior, and hospital data lacks information describing the crash itself, we decided to use interviews to acquire information.

There are several limitations in this research. Risk factors described in this article are primarily about hospitalization for motorcycle crashes; we did not assess non-fatal injuries that did not require a hospital stay of greater than one day, or fatal injuries. We only investigated hospitalized patients in the neurosurgery and orthopedic departments. Characteristics related to motorcycle crashes were self-reported, and no sources were available to validate the collected information. Additionally, information obtained from family members who may not have been on the scene would influence the reliability of the results. Behavioral factors, such as alcohol consumption, safeguard use, and unsafe behavior may have been under estimated. Also, not all patients were immediately investigated after admission, so there was memory bias. The actual levels of injury severity were vulnerable to misclassification because the judgment standard of injury severity might have been subjected to differences between doctors and between hospitals. According to the motorcycle injuries, there was heterogeneous in road users not just only motorcycle users.

In summary, we found that males, those without safeguards, morning and night hours, non-urban areas, collision of a motorcycle with a cycle, use of an ambulance, admission to the neurosurgery department, lack of traffic control, unobstructed traffic conditions, and poor visibility were associated with severe injuries in motorcycle crashes. This research highlights some problems, such as less helmet wearing in motorcyclists and cyclists, rural injuries being more serious than urban ones, and head injuries being the main diagnosis in severe injuries. The result of this research is predictable if the safety equipment is forced to be used, such as helmets; the traffic environment is improved, such as traffic flow, medical resources to injuries and deaths is seasonable, then traffic safety will be improved and accidents will be reduced. This research may provide impetus to prioritize programs that prevent and reduce motorcycle-related injuries in medium-sized cities and nationwide.
